# A Novel Synthetic Compound (E)-5-((4-oxo-4H-chromen-3-yl)methyleneamino)-1-phenyl-1H-pyrazole-4-carbonitrile Inhibits TNFα-Induced *MMP9* Expression via EGR-1 Downregulation in MDA-MB-231 Human Breast Cancer Cells

**DOI:** 10.3390/ijms21145080

**Published:** 2020-07-18

**Authors:** Munki Jeong, Euitaek Jung, Young Han Lee, Jeong Kon Seo, Seunghyun Ahn, Dongsoo Koh, Yoongho Lim, Soon Young Shin

**Affiliations:** 1 Department of Biological Sciences, Sanghuh College of Lifesciences, Koknkuk University, Seoul 05029, Korea; Jmk4919@naver.com (M.J.); mylife4sci@naver.com (E.J.); yhlee58@konkuk.ac.kr (Y.H.L.); 2 Cancer and Metabolism Institute, Konkuk University, Seoul 05029, Korea; yoongho@konkuk.ac.kr; 3 UNIST Central Research Facilities, Ulsan National Institute of Science and Technology, Ulsan 44919, Korea; jkseo6998@unist.ac.kr; 4 Department of Applied Chemistry, Dongduk Women’s University, Seoul 02748, Korea; mistahn321@naver.com (S.A.); dskoh@dongduk.ac.kr (D.K.); 5 Division of Bioscience and Biotechnology, BMIC, Konkuk University, Seoul 05029, Korea

**Keywords:** EGR-1, flavonoid, (E)-5-((4-oxo-4H-chromen-3-yl)methyleneamino)-1-phenyl-1H-pyrazole-4-carbonitrile, MDA-MB-231, MMP9, TNFα

## Abstract

Breast cancer is a common malignancy among women worldwide. Gelatinases such as matrix metallopeptidase 2 (MMP2) and MMP9 play crucial roles in cancer cell migration, invasion, and metastasis. To develop a novel platform compound, we synthesized a flavonoid derivative, (E)-5-((4-oxo-4H-chromen-3-yl)methyleneamino)-1-phenyl-1H-pyrazole-4-carbonitrile (named DK4023) and characterized its inhibitory effects on the motility and *MMP2* and *MMP9* expression of highly metastatic MDA-MB-231 breast cancer cells. We found that DK4023 inhibited tumor necrosis factor alpha (TNFα)-induced motility and F-actin formation of MDA-MB-231 cells. DK4023 also suppressed the TNFα-induced mRNA expression of *MMP9* through the downregulation of the TNFα-extracellular signal-regulated kinase (ERK)/early growth response 1 (EGR-1) signaling axis. These results suggest that DK4023 could serve as a potential platform compound for the development of novel chemopreventive/chemotherapeutic agents against invasive breast cancer.

## 1. Introduction

Breast cancer is a common malignancy among women worldwide [1]. Metastasis, the spread of primary tumor cells to different organs through the blood or lymphatic vessels, is a typical hallmark of all malignant tumors and is responsible for high mortality among patients with cancer. Breast cancer commonly spreads to the regional lymph nodes, bone, liver, lungs, and brain [2]. Thus, the control over the process of metastasis at the early stages is imperative for the successful prevention and treatment of cancer, including breast cancer.

Tumor cell invasion involves their migration and penetration into neighboring tissues. Lymphovascular invasion is, in general, the first stage of carcinogenic events that initiate tumor metastasis [3]. The initial step of invasion is characterized by the breakdown of the basement membrane, a sheet-like structure of the extracellular matrix secreted by the epithelium [4]. Type IV collagen is a major component of the basement membrane, and gelatinases such as matrix metallopeptidase 9 (MMP9, also known as gelatinase B and 92 kDa type IV collagenase) and MMP2 (also known as gelatinase A and 72 kDa type IV collagenase) are responsible for the breakdown of type IV collagen [5]. MMP2 and MMP9 expression is known to be highly upregulated in almost every metastatic cancer cell type [5,6], and elevated MMP9 level is associated with the high potential of metastasis in several human carcinomas, including breast cancer [6,7,8]. MMP9 expression has been observed in invasive mammary carcinomas, but not in carcinomas in situ or hyperplastic mammary glands [9]. Experimental metastasis studies have also shown that the downregulation of MMP9 expression in cancer cells by ribozyme could reduce tumor foci in the lungs of mice [10], consistent with the results observed in *Mmp2*- [10] or *Mmp9*-deficient mice [11]. These observations suggest that a therapeutic strategy to manipulate MMP2 or MMP9 expression could be potentially advantageous for the development of anti-metastatic agents.

Flavonoids have a common feature of C6-C3-C6 skeleton. One of the flavonoids, flavone is composed of 4H-chromen-4-one for C6-C3- and benzene ring for the -C6. Compounds carrying the 1-phenyl-1H-pyrazole moiety have been reported to exhibit antitumor activities [12] and induce apoptosis [13]. In addition, compounds with the 4H-chromen-4-one moiety are known to demonstrate BRAF kinase inhibitory activity [14], cytotoxicity against the breast cancer cell line MCF-7 [15], and inhibitory effects on the p53-mouse double minute 2 (MDM2) pathway [16]. Therefore, we synthesized a novel compound combining 1-phenyl-1H-pyrazole and 4H-chromen-4-one moieties to obtain (E)-5-((4-oxo-4H-chromen-3-yl)methyleneamino)-1-phenyl-1H-pyrazole-4-carbonitrile (termed as DK4023). When C2- and C3-position of flavonoids are substituted with benzene ring, they are classified as flavone and isoflavone, respectively. Since the C3-position is substituted with 5-(methyleneamino)-1-phenyl-1H-pyrazole-4-carbonitrile, DK4023 can be classified as isoflavone derivative. Among isoflavones, genistein and daidzein are known to inhibit TNFα-induced migration and invasion of human breast cancer cells by preventing the inhibition of NF-κB [17]. TNFα is a central inflammatory cytokine in the tumor microenvironment that promotes migration, invasion, and metastasis [18,19,20]. In the current study, we examined the inhibitory effects of DK4023 on TNFα-induced motility and invasive capability of the highly metastatic MDA-MB-231 breast cancer cells. In addition, we determined the inhibitory effect of DK4023 on TNFα-induced *MMP2* and *MMP9* mRNA expression.

## 2. Results and Discussion

### 2.1. Chemical Synthesis and Cytotoxicity of DK4023 against MDA-MB-231 Cells

**Scheme 1:**
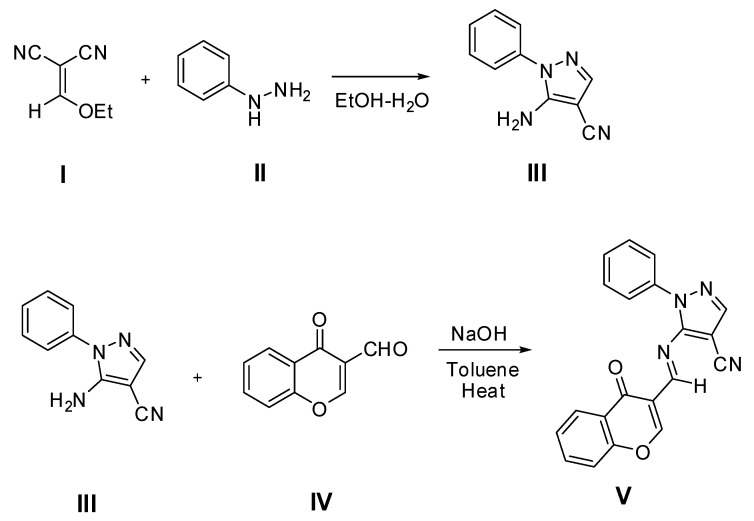
Synthesis of (E)-5-((4-oxo-4H-chromen-3-yl)methyleneamino)-1-phenyl-1H-pyrazole-4-carbonitrile (DK4023).

The synthesis of DK4023 was started from phenylhydrazine (**I**) and 2-(ethoxymethylidene) propanedinitrile (**II**). The resulting 5-amino-1-phenyl-1H-pyrazole-4-carbonitrile (**III**) was reacted with 4-oxo-4H-chromene-3-carbaldehyde (**IV**) to yield (E)-5-((4-oxo-4H-chromen-3-yl)methyleneamino)-1-phenyl-1H-pyrazole-4-carbonitrile (**V**; named DK4023) (Scheme 1).

**Figure 1:**
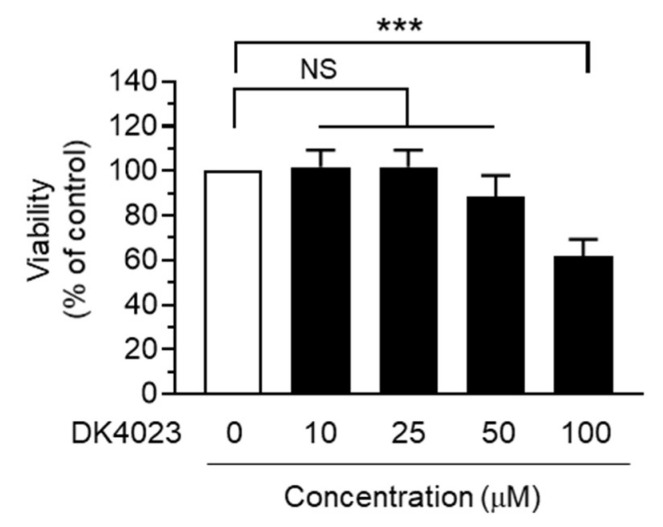
Cytotoxicity of DK4023 against MDA-MB-231 cells. Values are expressed as mean ± SD. *** *p* < 0.001, ^NS^ not significant (*p* > 0.200) by Sidak’s multiple comparison test (*n* = 3).

To determine the cytotoxicity of DK4023, we treated MDA-MB-231 human breast cancer cells with DK4023 (0, 10, 25, 50, and 100 μM) for 24 h. Cellular cytotoxicity was determined using the water-soluble 2-(2-methoxy-4-nitrophenyl)-3-(4-nitrophenyl)-5-(2,4-disulfophenyl)-2*H*-tetrazolium, monosodium salt (WST-8). As no significant cytotoxicity was observed at up to 50 μM DK4023 concentration (Figure 1), we used 25 and 50 μM concentrations for subsequent experiments.

### 2.2. Effect of DK4023 on the TNFα-Induced Migration of MDA-MB-231 Cells

The tumor mass is composed of tissue-resident fibroblasts, peripherally recruited immune cells, and endothelial cells of surrounding blood vessels, as well as cancer cell populations. The local environment around the tumor mass includes various growth factors and cytokines, which are collectively referred to as a tumor microenvironment [21]. It has been well characterized that the inflammatory tumor microenvironment is closely associated with tumor development and progression [22,23]. TNFα is a major proinflammatory cytokine that is released from many cell types, including cancer cells, immune cells, and fibroblasts, in the tumor microenvironment [20]. It has been demonstrated that TNFα increases the expression of other cytokines and chemokines, including IL-1, IL-6, CCL2, CXCL8, and CXCL12 [24], induces epithelial-to-mesenchymal transition (EMT), through the activation of NF-κB and AP1, and facilitates the invasion and metastasis of breast cancer cells [21,25].

**Figure 2:**
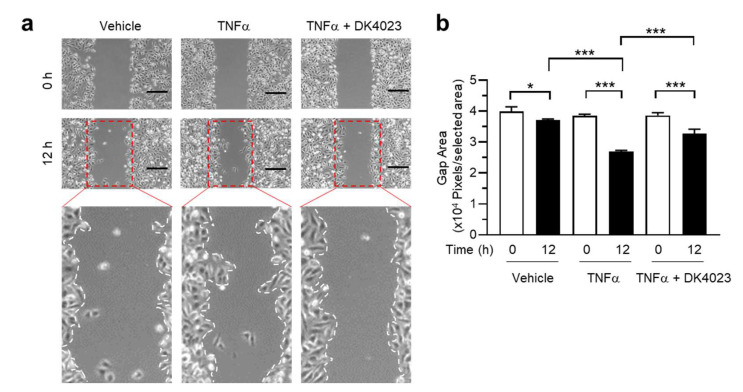
Effect of DK4023 on the migration of MDA-MB-231 cells. (**a**) Scratch wound-healing assay. DK4023 was pretreated for 30 min before addition of TNFα. Migration images were captured after 12 h of TNFα treatment. Box areas were enlarged underneath the image. Arrow indicates the elongated spindle shape of leader cells at the leading edge. Vehicle, phosphate-buffered saline. Scale bars, 500 μm. (**b**) A plot of the scratched gap area was expressed as the number of pixels in the selected boxes. Values were expressed as mean ± SD. * *p* = 0.021, *** *p* < 0.001 by Sidak’s multiple comparison test (*n* = 3).

A crucial feature of invasive and metastatic breast cancer cells is the increase in their motility. To evaluate whether DK4023 could modulate the motility of metastatic MDA-MB-231 cells, we used an in vitro scratch-wound healing assay and measured the thickness of the scratched area. After scratching a confluent monolayer, cells were treated with TNFα (10 ng/mL) or TNFα (10 ng/mL) plus DK4023 (25 and 50 μM) (Figure 2a). At 12 h post-scratching, the scratched area decreased following TNFα treatment as compared that observed after vehicle treatment. In contrast, the TNFα-induced closure of the scratched area was significantly suppressed in the presence of DK4023 (Figure 2b). As DK4023 did not exhibit cytotoxicity at concentrations around 50 μM (Figure 1), its inhibitory effect on the motility of MDA-MB-231 cells was not related to its cytotoxicity.

### 2.3. Effect of DK4023 on the Actin Reorganization of MDA-MB-231 Cells

**Figure 3:**
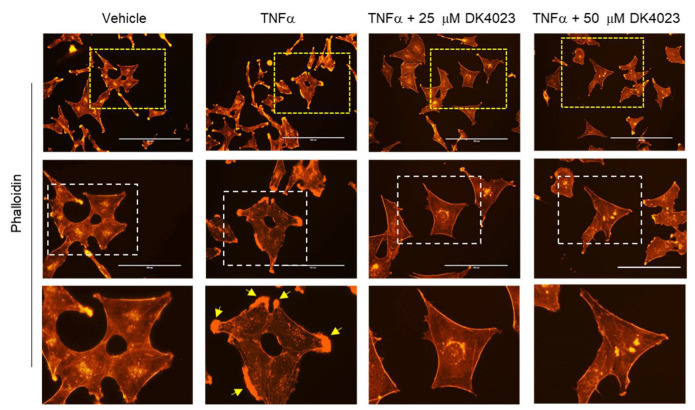
Effect of DK4023 on the actin rearrangement of MDA-MB-231 cells. Polymerized actin (F-actin) was stained with rhodamine-conjugated phalloidin. Box area was enlarged underneath the image. Arrows indicate lamellipodia. Bar size: top panels, 200 μm; middle panels, 100 μm.

As shown in Figure 3, DK4023 reduced the TNFα-induced branched structures of the leader cells at the edge (arrow). Monomeric globular actin (G-actin) is polymerized into filamentous actin (F-actin), which is known to build up higher-ordered structures such as stress fibers, lamellipodia, and filopodia during cell movement [26]. As the dynamic rearrangement of the actin cytoskeleton plays a crucial role in cell migration [27], we assessed whether DK4023 affects actin cytoskeletal rearrangement. We used rhodamine-conjugated phalloidin to stain F-actin and found that TNFα treatment stimulated cytoskeletal rearrangement, as evident from the formation of F-actin-rich protrusions that appeared like lamellipodia (arrows) at the cell periphery (Figure 3). After the treatment of cells with DK4023, the TNFα-induced F-actin-rich protrusions were substantially reduced. These data suggest that DK4023 prevents dynamic F-actin polymerization, resulting in the inhibition of cell motility.

### 2.4. Effect of DK4023 on the Expression of MMP9

**Figure 4:**
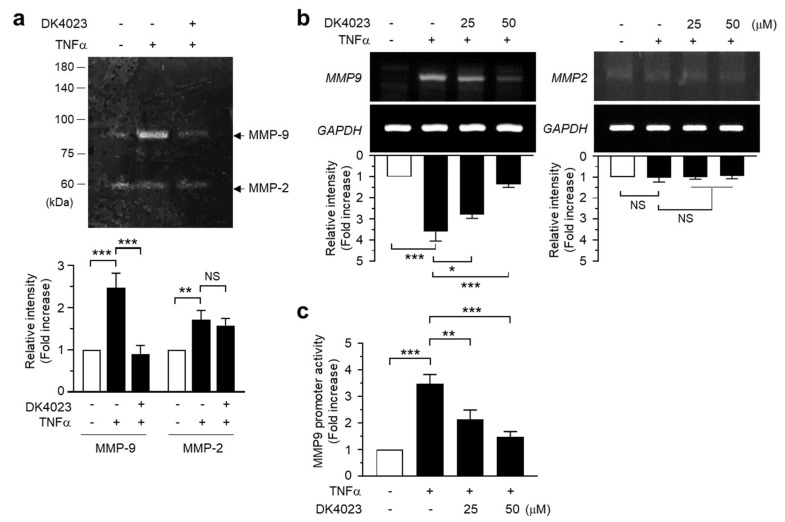
Effect of DK4023 on TNFα-induced MMP9 activation. Effect of DK4023 on TNFα-induced gelatinolytic activity determined by gelatin-gel zymography (**a**), *MMP9 and MMP2* mRNA expression examined by RT-PCR (**b**), and luciferase-based *MMP9* promoter-reporter activity (**c**). The full-length gel images are presented in Supplementary Figure S1. Relative band intensities were measured by ImageJ. Values are expressed as mean ± SD. * *p* = 0.016, ** *p* < 0.01, ***p < 0.001, NS, not significant (*p* > 0.5) by Sidak’s multiple comparison test (*n* = 3).

As gelatinases play a critical role in cell migration, invasion, and metastasis, we investigated whether DK4023 modulates gelatinase activation. Gelatinase activity was determined using gelatin-gel zymography. DK4023 reduced the density of TNFα-induced gelatinolytic white band at 92 kDa MMP9, but not that of the band at 72 kDa MMP2 (Figure 4a). To investigate whether DK4023-induced suppression of MMP9 activity was associated with *MMP9* downregulation, we determined the effect of DK4023 on the expression of *MMP9* mRNA using conventional reverse-transcription polymerase chain reaction (RT-PCR). We found that DK4023 dose-dependently inhibited TNFα-induced mRNA expression of *MMP9* but not *MMP2*, while glyceraldehyde 3-phosphate dehydrogenase (*GAPDH*) mRNA levels remained unchanged (Figure 4b). As TNFα induced MMP9 mRNA expression and proteolytic activity of MMP9 more efficiently than MMP2, we focused on the inhibitory effect of DK4023 on MMP9 expression in substantial experiments. To further evaluate the inhibitory effect of DK4023 on *MMP9* expression at the transcriptional level, we measured *MMP9* promoter activity using the pMMP9(−925/+13)-Luc luciferase-based promoter-reporter plasmid [28]. TNFα stimulated *MMP9* promoter activity by 3.47 ± 0.351-fold, but this effect was significantly suppressed by 2.13 ± 0.351- and 1.47 ± 0.208-fold in the presence of 25 and 50 μM DK4023, respectively (Figure 4c). Thus, DK4023 inhibits *MMP9* mRNA expression.

### 2.5. Effect of DK4023 on the TNFα-Induced Invasion of MD-MB-231 Cells

**Figure 5:**
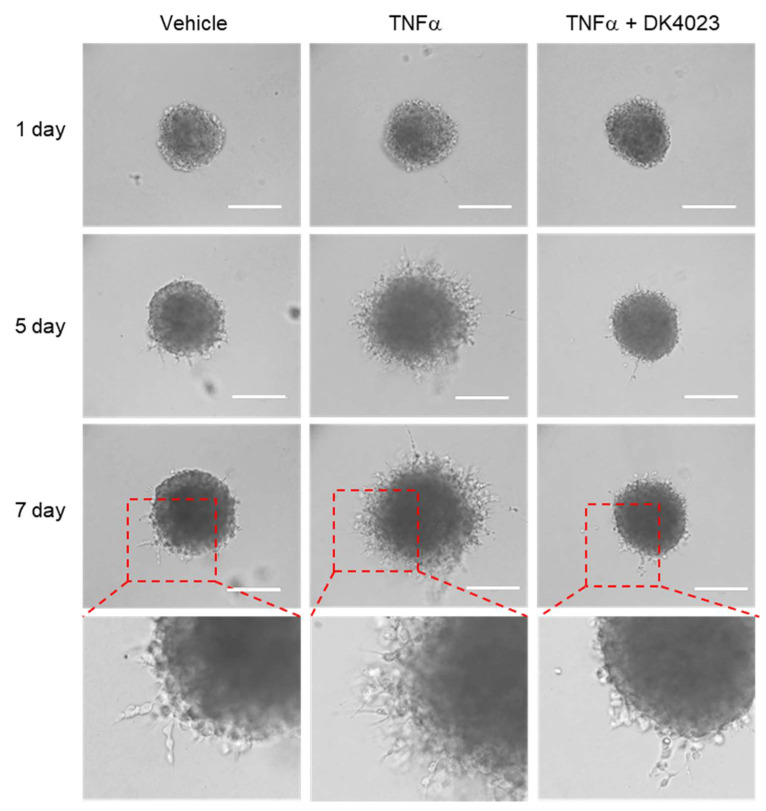
Effect of DK4023 on the invasion of MDA-MB-231 cells. Schematic representation of three-dimensional spheroid formation and protrusion of MDA-MB-231 cells (left). MDA-MB-231 cell spheroids in the extracellular matrix were treated with vehicle (DMSO) or 10 ng/mL TNFα for 7 days in the presence or absence of 25 μM DK4023. The morphology of the invasive protrusions was captured with an EVOS FL Auto Cell Imaging System. Scale bars, 400 μm. The boxed areas are enlarged.

MMP9 plays a critical role in the invasion of tumor cells through the degradation of extracellular matrix proteins [5]. To investigate the effect of DK4023 on the invasive capability of MDA-MB-231 cells, we used a matrigel-based three-dimensional (3-D) spheroid culture model that closely resembles the situation inside the in vivo tumor mass. Up to 1 day, the cells within the spheroid were noninvasive and remained as cell aggregates. (Figure 5, top panels). Following 5 days of culture, invasive cells could be detected in vehicle-treated spheroid (Figure 5, left lane). Following treatment with 10 ng/mL TNFα, the sizes of spheroids were bigger than vehicle-treated groups, and the cells efficiently invaded the surrounding matrix as spindle-like protrusions (Figure 5, middle lane). However, in the presence of DK4023, TNFα-induced invasive capability was substantially attenuated (Figure 5, right lane). These results suggest that DK4023 inhibits TNFα-induced tumor cell invasion through the downregulation of MMP9 expression.

### 2.6. Effect of DK4023 on the TNFα-Induced Activation of Nuclear Factor-Kappa B (NF-κB) Pathway

**Figure 6:**
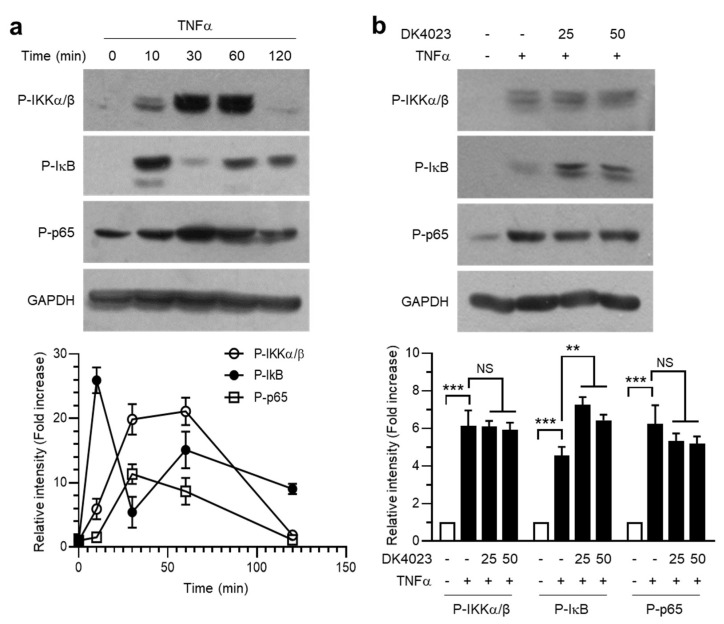
Effect of DK4023 on the TNFα-induced activation of NF-κB pathway. (**a**) The TNFα-induced phosphorylation of IKKα/β on Ser-180/181, IκB on Ser-32, and RelA/p65 on Ser-536 were determined by immunoblot analysis. (**b**) The effect of DK4023 on the TNFα-induced phosphorylation of NF-κB pathway proteins was determined by immunoblotting. Relative band intensities were measured by ImageJ. Values are expressed as mean ± SD. *** *p* < 0.001, ** *p* < 0.01, ^NS^ not significant (*p* > 0.05) by Sidak’s multiple comparison test (*n* = 3). The full-length blot images are presented in Supplementary Figure S2.

NF-κB is a well-known transcription factor involved in the regulation of TNFα-induced *MMP9* expression [29,30,31,32]. In unstimulated cells, RelA/p65 and p50, which are the most abundant forms of NF-κB heterodimers, are inhibited by IκB in the cytoplasm. Upon exposure of cells to TNFα, IκB kinase (IKK) gets activated and phosphorylates IκB, subsequently resulting in the degradation of IκB through a ubiquitin-mediated proteolytic pathway [33]. We observed that TNFα enhanced the phosphorylation of IKK and RelA/p65 in a time-dependent manner, while IκB phosphorylation level increased within 10 min, then rapidly decreased, and slowly recovered thereafter in MDA-MB-231 cells (Figure 6a). To determine whether DK4023 modulates the NF-κB pathway, we pretreated MDA-MB-231 cells with DK4023 and measured the TNFα-induced phosphorylation status of three key proteins, IKK, IκB, and RelA/p65. DK4023 failed to significantly decrease the TNFα-induced phosphorylation of IKKα/β or RelA/p65 but could significantly increase the phosphorylation of IκB (Figure 6b). These data suggest that DK4023 may affect the NF-κB pathway through the stabilization of IκB, which could be necessary but not sufficient to downregulate *MMP9* expression.

### 2.7. Effect of DK4023 on the TNFα-Induced Expression of Early Growth Response-1 (EGR-1)

**Figure 7:**
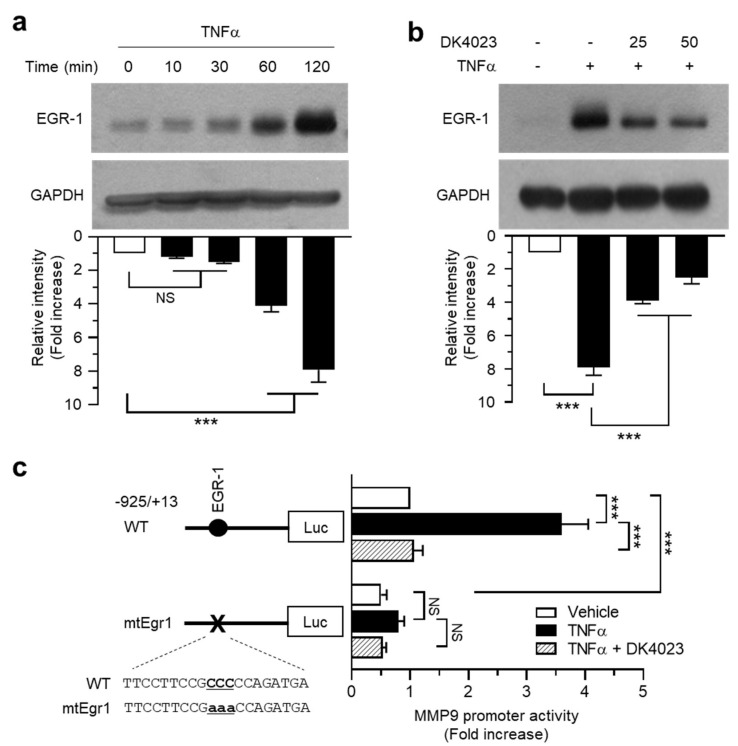
Effect of DK4023 on the TNFα-induced expression of EGR-1. (**a**) The TNFα-induced accumulation of EGR-1 was determined by immunoblotting. (**b**) Inhibitory effect of DK4023 on TNFα-induced EGR-1 accumulation was determined by immunoblotting. Relative band intensities were measured by ImageJ. The full-length blot images are presented in Supplementary Figure S3. (**c**) The effect of the mutation of the EGR-1-binding element in *MMP9* promoter-reporter construct, pMMP9-Luc(−925/+13), on the TNFα-induced activation of *MMP9* promoter was determined by luciferase reporter activity. WT, wild-type; mtEgr1, mutation of the EGR-1-binding sequence. Values were expressed as mean ± SD. *** *p* < 0.001, ^NS^ not significant (*p* > 0.05) by Dunnett’s (**a**,**b**) or Sidak’s multiple comparison test (**c**).

EGR-1 is a Cys_2_His_2_-type zinc-finger transcription factor induced by mitogenic stimulation and DNA damage signals [34]. EGR-1 is known to directly bind to the EGR-1-binding element within the *MMP9* gene promoter and transactivate the *MMP9* promoter activity upon TNFα stimulation in HeLa cervical cancer cells [28]. In MDA-MB-231 cells, TNFα increased the level of EGR-1 protein in a time-dependent manner (Figure 7a). However, DK4023 decreased the TNFα-induced expression of EGR-1 in a dose-dependent manner (Figure 7b). To evaluate the role of EGR-1 in DK4023-induced *MMP9* suppression, we used the wild-type *MMP9* promoter construct and a site-directed mutant construct obtained by disrupting the EGR-1-binding site (EBS) at *MMP9* promoter-reporter. TNFα stimulated *MMP9* promoter activation in the wild-type construct, but its effect was lost following the disruption of EBS (Figure 7c). DK4023 treatment inhibited the TNFα-induced *MMP9* promoter activation in the wild-type construct. These data suggest that EGR-1 is critical for mediating the TNFα-induced activation of *MMP9* promoter and that EGR-1 downregulation by DK4023 is associated with the suppression of TNFα-induced *MMP9* promoter activation.

### 2.8. Effect of DK4023 on the TNFα-Induced Activation of Mitogen-Activated Protein Kinase (MAPK) Pathway

**Figure 8:**
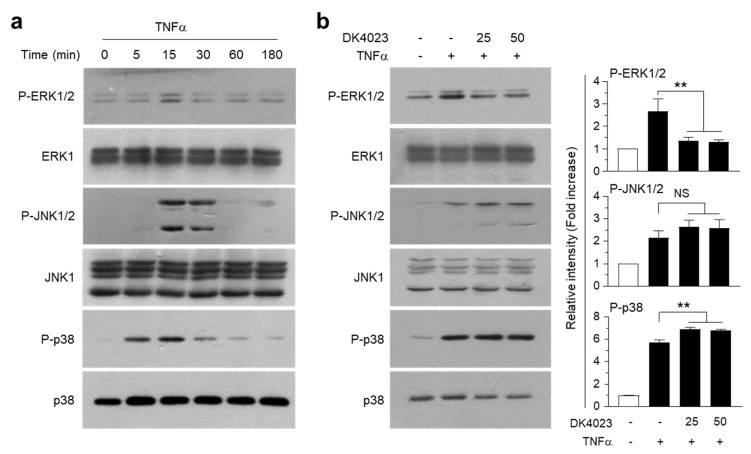
Effect of DK4023 on the TNFα-induced activation of MAPK pathways. (**a**) The TNFα-induced phosphorylation of ERK1/2 on Thr-201/Tyr-204, JNK1/2 on Thr-183/Tyr-185, and p38 on Thr-180/Tyr-182 were determined by immunoblotting. (**b**) The effect of DK4023 on the TNFα-induced phosphorylation of MAPKs was determined by immunoblotting. Relative band intensities were measured by ImageJ. Values were expressed as mean ± SD. ** *p* < 0.01, ^NS^ not significant (*p* > 0.05) by Dunnett’s multiple comparison test (*n* = 3). The full-length blot images are presented in Supplementary Figure S4.

MAPK signaling pathway plays an essential role in mediating the TNFα-induced expression of EGR-1 in MDA-MB-231 cells [35]. We investigated if DK4023 modulates MAPK pathways by evaluating its effects on the TNFα-induced phosphorylation of three key MAPKs, extracellular signal-regulated kinase 1/2 (ERK1/2), c-Jun N-terminal kinase 1/2 (JNK1/2), and p38 kinase, in MDA-MB-231 cells using phospho-specific antibodies. We found that the phosphorylation of ERK1/2 on Thr-201/Tyr-204, JNK1/2 on Thr-183/Tyr-185, and p38 kinase on Thr-180/Tyr-182 increased within 15 min of TNFα treatment as compared to that observed under unstimulated condition (Figure 8a). Under these conditions, pretreatment of MDA-MB-231 cells with DK4023 significantly reduced (*p* < 0.01, *n* = 3) the levels of TNFα-induced ERK1/2, whereas did not affect or slightly increased the phosphorylation of JNK1/2 and p38 kinase (Figure 8b). These data suggest that DK4023 reduces TNFα-induced *MMP9* expression by selectively inhibiting the ERK1/2 and EGR-1 signaling axis.

## 3. Materials and Methods

### 3.1. Cells and Chemicals

MDA-MB-231 human breast cancer cells were obtained from the American Type Culture Collection (ATCC, Rockville, MD, USA) and cultured in Dulbecco’s modified Eagle’s medium (Corning Cellgro, Manassas, VA, USA) supplemented with 10% (v/v) heat-inactivated fetal bovine serum (Corning Cellgro). TNFα was purchased from Sigma-Aldrich (Saint Louis, MO, USA), and Pierce BCA Protein Assay Reagent was supplied by Thermo Scientific (Rockford, IL, USA). The Firefly and *Renilla* Dual-Glo^™^ Luciferase Assay System was procured from Promega (Madison, WI, USA), and nitrocellulose membrane, from Bio-Rad Laboratories (Hercules, CA, USA).

### 3.2. Chemical Synthesis

Commercially available phenylhydrazine (**I**, 1.10 g, 10 mmol) and 2-(ethoxymethylidene) propanedinitrile (**II**, 10 mmol, 1.22 g) were dissolved in 20 mL of an ethanol-water (1:3) solution, and the resulting mixture was stirred at 25 °C for 20 h to obtain an orange color solid. The precipitate was filtered and washed with cold ethanol. The resulting solid of **III**, 5-amino-1-phenyl-1H-pyrazole-4-carbonitrile, was dried and used for the next reaction without any further purification. In brief, 5 mL of toluene solvent was mixed with equimolar amounts of 5-amino-1-phenyl-1H-pyrazole-4-carbonitrile (**III**, 60 mg, 0.3 mmol) and 4-oxo-4H-chromene-3-carbaldehyde (**IV**, 51 mg, 0.3 mmol), and the mixture was treated with a catalytic amount of sodium hydroxide (NaOH). The reaction mixture was stirred at 75 °C for 5 h. After reaction completion, the mixture was cooled to room temperature and the solvent was evaporated under the vacuum. The residues were purified with medium-pressure chromatography to obtain an analytically pure compound of **V** (named DK4023). The structure of DK4023 was verified using nuclear magnetic resonance (NMR) spectroscopy and high-resolution electrospray ionization mass spectrometry (HR-ESI-MS) as described previously [36]. Its spectroscopic data are summarized as follows: IR (cm^−1^) 3112.55(N-H), 2216.77(-CN), 1657.62(conj. C=O); ^1^H NMR (500 MHz, DMSO-d6) δ 9.23 (d, 1H, *J* = 0.5 Hz), 9.04 (m, 1H), 8.32 (s, 1H), 8.17 (dd, 1H, *J* = 8.0, 1.5 Hz), 7.90 (m, 1H), 7.76 (dd, 1H, *J* = 8.5, 0.7 Hz), 7.71 (m, 2H), 7.60 (m, 1H), 7.55 (m, 2H), 7.46 (m, 1H); ^13^C NMR (125 MHz, DMSO-d6) δ 174.64 161.00, 160.03, 155.65, 152.23, 142.90, 137.64, 135.18, 129.09, 128.13, 126.75, 125.35, 124.05, 123.64, 118.88, 118.77, 113.86, 81.23, 48.56.; HR-ESI-MS (*m*/*z*): Calcd. for C_20_H_12_N_4_O_2_ [M+H]^+^: 341.1039; found 341.1036. The raw spectra, including IR, ^1^H-NMR, ^13^C-NMR, and HR-ESI-Mass spectrum, are provided as a Supplementary Figure S5.

### 3.3. Cytotoxicity Assay

MDA-MB-231 cells cultured in 96-well plates (2 × 10^3^ cells/well) were treated with different concentrations (10–100 μM) of DK4023 for 24 h. Cell viability was measured by the Cell Counting Kit-8 (Dojindo Molecular Technologies, Gaithersburg, MD) assay using the water-soluble tetrazolium salt WST-8, as previously described [37]. The absorbance was read at 540 nm using a microplate reader (Molecular Devices Corp., Menlo Park, CA).

### 3.4. Cell Migration Assay

Cell motility was examined using an in vitro scratch-wound healing assay, as previously described [19]. In brief, MDA-MB-231 cells were cultured in six-well plates and allowed to reach confluency. A scratch wound was made with a pipette tip on the confluent cell layer, which was then pretreated with either vehicle (DMSO) or 25 μM DK4023. After 30 min, cells were treated with either vehicle (PBS) or 10 ng/mL TNFα. Gap closure was imaged at 12 h post-scratching using an EVOS FL Auto Cell Imaging System (Life Technologies, Carlsbad, CA, USA) and the area of the closed gap was quantified using ImageJ version 1.52a (http://imagej.nih.gov/ij/; Center for Information Technology, National Institute of Health, Bethesda, MA, USA).

### 3.5. Actin Rearrangement

MDA-MB-231 cells were pretreated with either vehicle (DMSO) or DK4023 (25 or 50 μM). After 30 min, cells were treated with either vehicle (PBS) or TNFα (10 ng/mL) for 24 h, and then fixed in 4% paraformaldehyde and permeabilized in 0.3% Triton X-100. Actin polymerization was examined using the rhodamine/phalloidin-based F-Actin Visualization Biochem Kit (Cytoskeleton, Inc.; Denver, CO, USA) [38]. Images were captured using EVOS FL Auto Cell Imaging System.

### 3.6. In-Gel Gelatinase Activity Assay

Gelatin zymography was carried out using the Novex Zymogram gel system (Novex, San Diego, CA), as previously described [39]. In brief, MDA-MB-231 cells (3 × 10^5^) were cultured in a serum-free medium in 24-well plates for 24 h and then pretreated with either vehicle (DMSO) or DK4023 (25 or 50 μM). After 30 min, cells were treated with either vehicle (PBS) or TNFα (10 ng/mL). After 24 h, the culture medium was collected following the removal of cell debris and mixed with a non-reducing sample buffer (62.5 mM Tris-HCl [pH 6.8], 4% SDS, 25% glycerol, and 0.01% bromophenol blue). Equal amounts of each sample were electrophoresed using Novex pre-cast gels (10% acrylamide-0.1% gelatin). After electrophoresis, gels were equilibrated by incubation first in Novex Zymogram renaturation buffer for 30 min and then overnight in Zymogram developing buffer at 37 °C. Destaining was performed in methanol/acetic acid/distilled water (25:7:68, by volume) until gelatinolytic white bands appeared on the blue background. Relative gelatinolytic activities of MMP2 and MMP9 were determined by quantifying the white band intensities using ImageJ version 1.52a.

### 3.7. RT-PCR

The experimental procedures for total RNA isolation, cDNA synthesis, and PCR conditions are described elsewhere [39]. Briefly, cDNA was synthesized using 1 μg of total RNA and subjected to PCR using the following gene-specific primers: *MMP9* (forward) 5′-agattccaaacctttgag-3′ and (reverse) 5′-ggccttggaagatgaatg-3′; and *GAPDH* (forward) 5′-acccactcctccacctttg-3′ and (reverse) 5′-ctcttgtgctcttgctggg-3′. PCR conditions were as follows: denaturation, 94 °C for 30 s; annealing, 55 °C for 30 s; elongation, 72 °C for 1 min. The amplified products were subjected to electrophoresis on a 1% agarose gel. The relative *MMP9* mRNA level was quantitated after normalizing to *GAPDH* mRNA level using ImageJ version 1.52a.

### 3.8. MMP9 Promoter-Reporter Assay

The generation of the *MMP9* promoter construct pMMP9-Luc(−925/+13) and the mutated construct for EGR-1 binding site, pMMP9-Luc(−925/+13)mtEgr1, was described elsewhere [28]. MDA-MB-231 cells cultured on 12-well plates were transfected with 0.1 μg of MMP9 promoter-reporter constructs along with 25 ng of the pRL-null plasmid encoding *Renilla* luciferase to monitor transfection efficiency. At 48 h post-transfection, cells were pretreated with either vehicle (DMSO) or DK4023 (25 or 50 μM). After 30 min, cells were treated with either vehicle (PBS) or TNFα (10 ng/mL). After 8 h, cells were collected and the firefly luciferase activity was measured using a Centro LB960 dual luminometer (Berthold Technologies) as previously described [28].

### 3.9. 3-D Spheroid Culture and Invasion Assay

The 3-D invasion assay was carried out using the Cultrex 3-D Spheroid Cell Invasion Assay kit (Trevigen, Inc., Gaithersburg, MD, USA), as previously described [39]. Briefly, after forming spheroids of MDA-MB-231 cells, spheroids were embedded in matrigel-based extracellular matrix components and treated with or without 10 ng/mL TNFα in the presence or absence of 25 μM DK4023 for 7 days. Invasive protrusions into the surrounding extracellular matrix were visualized with an EVOS^®^ FL Auto Cell Imaging System (Life Technologies).

### 3.10. Immunoblot Analysis

Immunoblotting was performed as previously described [28]. In brief, MDA-MB-231 cells were lysed in a buffer comprising 20 mM HEPES (pH 7.2), 1% Triton X-100, 10% glycerol, 150 mM sodium hydroxide (NaCl), 10 μg/mL leupeptin, and 1 mM phenylmethylsulfonyl fluoride and electrophoresed on 10% SDS polyacrylamide gel electrophoresis (PAGE) gels. The separated protein bands were transferred onto nitrocellulose membranes, which were incubated with appropriate primary and horseradish peroxidase-conjugated secondary antibodies. The blots were then developed using an Amersham ECL Western Blotting Detection Kit (GE Healthcare Life Science, Chicago, IL, USA).

### 3.11. Statistical Analysis

Statistical significance was analyzed using GraphPad PRISM software version 8.2.1 (GraphPad Software Inc., La Jolla, CA, USA). A value of *p* < 0.05 was considered significant.

## 4. Conclusions

The present study identified a novel synthetic isoflavone derivative, DK4023, as an anti-invasive agent against metastatic breast cancer cells. Natural isoflavones, such as genistein and daidzein, are known to inhibit TNFα-induced migration and invasion of human breast cancer cells by preventing the inhibition of NF-κB [17]. This study demonstrated that DK4023 inhibited TNFα-induced migration and lamellipodium formation of highly metastatic MDA-MB-231 cells. Our findings also show that DK4023 suppressed TNFα-induced expression of *MMP9* mRNA through the downregulation of the ERK1/2-mediated expression of EGR-1, independently of NF-κB. More importantly, DK4023 substantially attenuated the invasive capability of MDA-MB-231 breast cancer cells. Although additional in vivo studies are warranted to validate the clinical efficacy of DK4023 in a metastasis animal model, we propose that DK4023 can serve as a promising agent to target the TNFα-ERK1/2 MAPK-EGR-1-MMP9 signaling pathway for the development of a chemopreventive or chemotherapeutic adjuvant, which can be used in combination with conventional chemotherapy, radiotherapy, or immunotherapy against metastatic cancers, particularly breast cancer.

## Supplementary Materials

The following are available online at https://www.mdpi.com/1422-0067/21/14/5080/s1.

## Abbreviations

DK4023	(E)-5-((4-oxo-4H-chromen-3-yl)methyleneamino)-1-phenyl-1H-pyrazole-4-carbonitrile
EBS	EGR-1-binding site
EGR-1	Early growth response-1
ERK	Extracellular signal-regulated kinase
GAPDH	Glyceraldehyde 3-phosphate dehydrogenase
IκB	Inhibitor of nuclear factor-κB
IKK	IκB kinase
JNK	c-Jun N-terminal kinase
MAPK	Mitogen-activated protein kinase
MMP	Matrix metallopeptidase
NF-κB	Nuclear factor kappa B
RelA	v-rel Avian reticuloendotheliosis viral oncogene homolog A
RT-PCR	Reverse-transcription polymerase chain reaction
TNFα	Tumor necrosis factor alpha

## References

[1] Jemal A., Bray F., Center M.M., Ferlay J., Ward E., Forman D. (2011). Global cancer statistics. CA Cancer J. Clin..

[2] Lacroix M. (2006). Significance, detection and markers of disseminated breast cancer cells. Endocr. Relat. Cancer.

[3] Kurozumi S., Joseph C., Sonbul S., Alsaeed S., Kariri Y., Aljohani A., Raafat S., Alsaleem M., Ogden A., Johnston S.J. (2019). A key genomic subtype associated with lymphovascular invasion in invasive breast cancer. Br. J. Cancer.

[4] Gatseva A., Sin Y.Y., Brezzo G., Van Agtmael T. (2019). Basement membrane collagens and disease mechanisms. Essays Biochem..

[5] Egeblad M., Werb Z. (2002). New functions for the matrix metalloproteinases in cancer progression. Nat. Rev. Cancer.

[6] Morini M., Mottolese M., Ferrari N., Ghiorzo F., Buglioni S., Mortarini R., Noonan D.M., Natali P.G., Albini A. (2000). The alpha 3 beta 1 integrin is associated with mammary carcinoma cell metastasis, invasion, and gelatinase B (MMP-9) activity. Int. J. Cancer.

[7] Hidalgo M., Eckhardt S.G. (2001). Development of matrix metalloproteinase inhibitors in cancer therapy. J. Natl. Cancer Inst..

[8] Jones J.L., Shaw J.A., Pringle J.H., Walker R.A. (2003). Primary breast myoepithelial cells exert an invasion-suppressor effect on breast cancer cells via paracrine down-regulation of MMP expression in fibroblasts and tumour cells. J. Pathol..

[9] Kupferman M.E., Fini M.E., Muller W.J., Weber R., Cheng Y., Muschel R.J. (2000). Matrix metalloproteinase 9 promoter activity is induced coincident with invasion during tumor progression. Am. J. Pathol..

[10] Itoh T., Tanioka M., Yoshida H., Yoshioka T., Nishimoto H., Itohara S. (1998). Reduced angiogenesis and tumor progression in gelatinase A-deficient mice. Cancer Res..

[11] Itoh T., Tanioka M., Matsuda H., Nishimoto H., Yoshioka T., Suzuki R., Uehira M. (1999). Experimental metastasis is suppressed in MMP-9-deficient mice. Clin. Exp. Metastasis.

[12] Insuasty B., Tigreros A., Orozco F., Quiroga J., Abonia R., Nogueras M., Sanchez A., Cobo J. (2010). Synthesis of novel pyrazolic analogues of chalcones and their 3-aryl-4-(3-aryl-4,5-dihydro-1H-pyrazol-5-yl)-1-phenyl-1H-pyrazole derivatives as potential antitumor agents. Bioorg. Med. Chem..

[13] Mellini P., Marrocco B., Borovika D., Polletta L., Carnevale I., Saladini S., Stazi G., Zwergel C., Trapencieris P., Ferretti E. (2018). Pyrazole-based inhibitors of enhancer of zeste homologue 2 induce apoptosis and autophagy in cancer cells. Philos. Trans. R. Soc. Lond. B Biol. Sci..

[14] Lu X., Dong G., Zheng Y., Zhang C., Qiu Y., Lua T., Zhou X. (2017). Synthesis and Anticancer Study of Novel 4H-Chromen Derivatives. Anticancer Agents Med. Chem..

[15] Singh A.K., Saxena G., Sahabjada S., Arshad M. (2017). Synthesis, characterization and biological evaluation of ruthenium flavanol complexes against breast cancer. Spectrochim. Acta A Mol. Biomol. Spectrosc..

[16] Bhatia R.K., Singh L., Garg R., Kaur M., Yadav M., Madan J., Kancherla S., Pissurlenkar R.R.S., Coutinho E.C. (2020). Novel p-Functionalized Chromen-4-on-3-yl Chalcones Bearing Astonishing Boronic Acid Moiety as MDM2 Inhibitor: Synthesis, Cytotoxic Evaluation and Simulation Studies. Med. Chem..

[17] Valachovicova T., Slivova V., Bergman H., Shuherk J., Sliva D. (2004). Soy isoflavones suppress invasiveness of breast cancer cells by the inhibition of NF-kappaB/AP-1-dependent and -independent pathways. Int. J. Oncol..

[18] Balkwill F. (2006). TNF-alpha in promotion and progression of cancer. Cancer Metastasis Rev..

[19] Sethi G., Sung B., Aggarwal B.B. (2008). TNF: A master switch for inflammation to cancer. Front. Biosci..

[20] Balkwill F. (2009). Tumour necrosis factor and cancer. Nat. Rev. Cancer.

[21] Lim B., Woodward W.A., Wang X., Reuben J.M., Ueno N.T. (2018). Inflammatory breast cancer biology: The tumour microenvironment is key. Nat. Rev. Cancer.

[22] Ham M., Moon A. (2013). Inflammatory and microenvironmental factors involved in breast cancer progression. Arch. Pharm. Res..

[23] Mantovani A., Allavena P., Sica A., Balkwill F. (2008). Cancer-related inflammation. Nature.

[24] Krones-Herzig A., Adamson E., Mercola D. (2003). Early growth response 1 protein, an upstream gatekeeper of the p53 tumor suppressor, controls replicative senescence. Proc. Natl. Acad. Sci. USA.

[25] Ham B., Fernandez M.C., D’Costa Z., Brodt P. (2016). The diverse roles of the TNF axis in cancer progression and metastasis. Trends Cancer Res..

[26] Merino F., Pospich S., Raunser S. (2020). Towards a structural understanding of the remodeling of the actin cytoskeleton. Semin. Cell Dev. Biol..

[27] Schaks M., Giannone G., Rottner K. (2019). Actin dynamics in cell migration. Essays Biochem..

[28] Shin S.Y., Kim J.H., Baker A., Lim Y., Lee Y.H. (2010). Transcription factor Egr-1 is essential for maximal matrix metalloproteinase-9 transcription by tumor necrosis factor alpha. Mol. Cancer Res..

[29] Esteve P.O., Chicoine E., Robledo O., Aoudjit F., Descoteaux A., Potworowski E.F., St-Pierre Y. (2002). Protein kinase C-zeta regulates transcription of the matrix metalloproteinase-9 gene induced by IL-1 and TNF-alpha in glioma cells via NF-kappa B. J. Biol Chem..

[30] Huang L., Lin H., Chen Q., Yu L., Bai D. (2019). MPPa-PDT suppresses breast tumor migration/invasion by inhibiting Akt-NF-kappaB-dependent MMP-9 expression via ROS. BMC Cancer.

[31] Wang J., Li S., Li X., Li B., Li Y., Xia K., Yang Y., Aman S., Wang M., Wu H. (2019). Circadian protein BMAL1 promotes breast cancer cell invasion and metastasis by up-regulating matrix metalloproteinase9 expression. Cancer Cell Int..

[32] Yang H.L., Thiyagarajan V., Shen P.C., Mathew D.C., Lin K.Y., Liao J.W., Hseu Y.C. (2019). Anti-EMT properties of CoQ0 attributed to PI3K/AKT/NFKB/MMP-9 signaling pathway through ROS-mediated apoptosis. J. Exp. Clin. Cancer Res..

[33] Taniguchi K., Karin M. (2018). NF-kappaB, inflammation, immunity and cancer: Coming of age. Nat. Rev. Immunol..

[34] Gashler A., Sukhatme V.P. (1995). Early growth response protein 1 (Egr-1): Prototype of a zinc-finger family of transcription factors. Prog. Nucleic Acid Res. Mol. Biol..

[35] Shin S.Y., Lee J.M., Lim Y., Lee Y.H. (2013). Transcriptional regulation of the growth-regulated oncogene alpha gene by early growth response protein-1 in response to tumor necrosis factor alpha stimulation. Biochim. Biophys. Acta.

[36] Koh D., Jung Y., Ahn S., Mok K.H., Shin S.Y., Lim Y. (2017). Synthesis and structure elucidation of polyphenols containing the N’-methyleneformohydrazide scaffold as aurora kinase inhibitors. Magn. Reson. Chem..

[37] Shin S.Y., Lee J., Park J., Lee Y., Ahn S., Lee J.H., Koh D., Lee Y.H., Lim Y. (2019). Design, synthesis, and biological activities of 1-aryl-(3-(2-styryl)phenyl)prop-2-en-1-ones. Bioorg. Chem..

[38] Lee D.Y., Lee D.H., Jung J.Y., Koh D., Kim G.S., Ahn Y.S., Lee Y.H., Lim Y., Shin S.Y. (2016). A synthetic chalcone derivative, 2-hydroxy-3′,5,5′-trimethoxychalcone (DK-139), suppresses the TNFalpha-induced invasive capability of MDA-MB-231 human breast cancer cells by inhibiting NF-kappaB-mediated GROalpha expression. Bioorg. Med. Chem. Lett..

[39] Shin S.Y., Kim C.G., Jung Y.J., Lim Y., Lee Y.H. (2016). The UPR inducer DPP23 inhibits the metastatic potential of MDA-MB-231 human breast cancer cells by targeting the Akt-IKK-NF-kappaB-MMP-9 axis. Sci. Rep..

