# Supplementary material for: A Novel Synthetic Compound (E)-5-((4-oxo-4H-chromen-3-yl)methyleneamino)-1-phenyl-1H-pyrazole-4-carbonitrile Inhibits TNFα-Induced MMP9 Expression via EGR-1 Downregulation in MDA-MB-231 Human Breast Cancer Cells
Source: Int J Mol Sci. 2020 Jul 18;21(14):5080. doi: 10.3390/ijms21145080 (PMC7404000; doi:10.3390/ijms21145080)

DK4023 IR spectrum

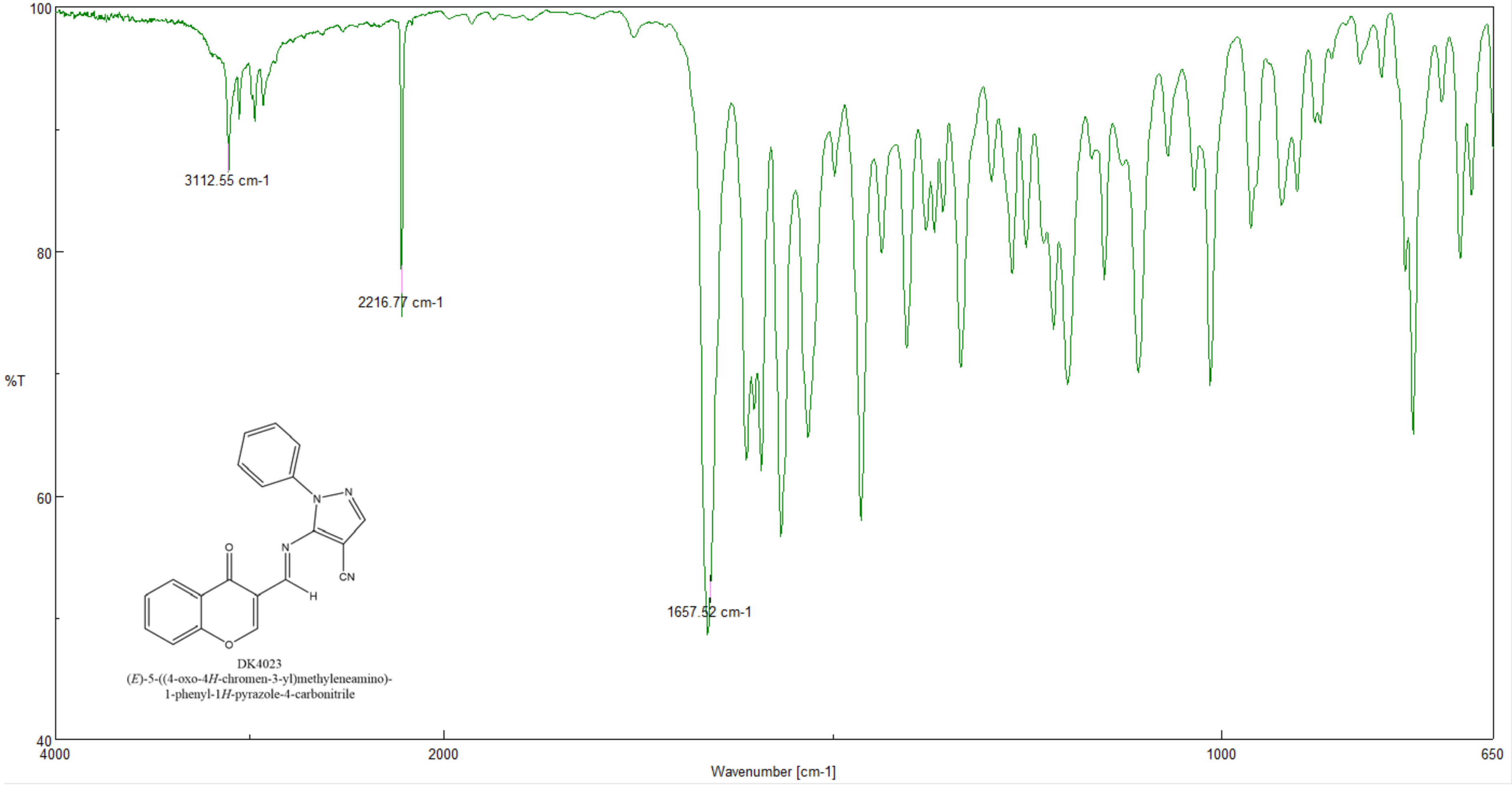

DK4023 ^{1}H-NMR spectrum

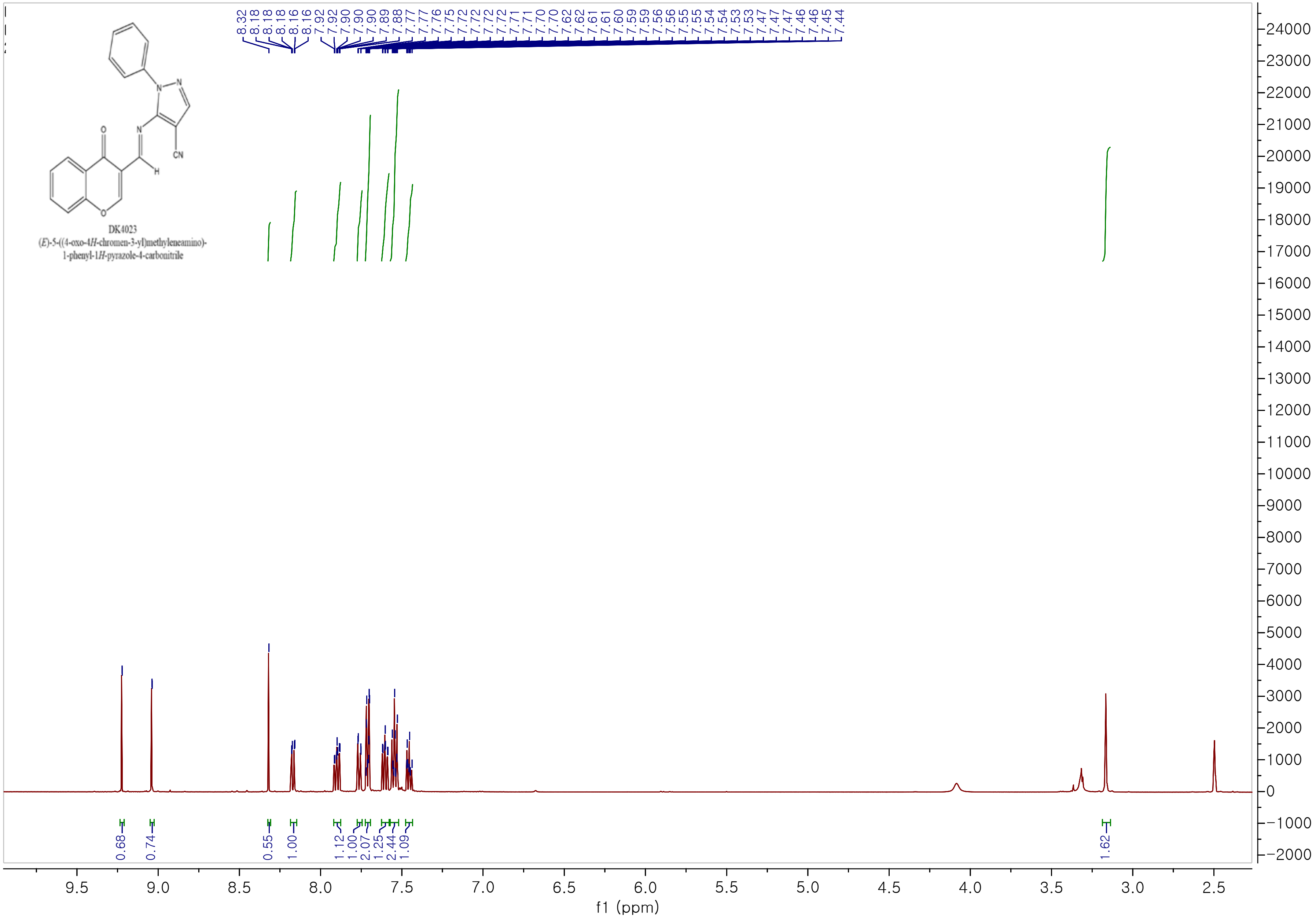

DK4023 ^{13}C-NMR spectrum

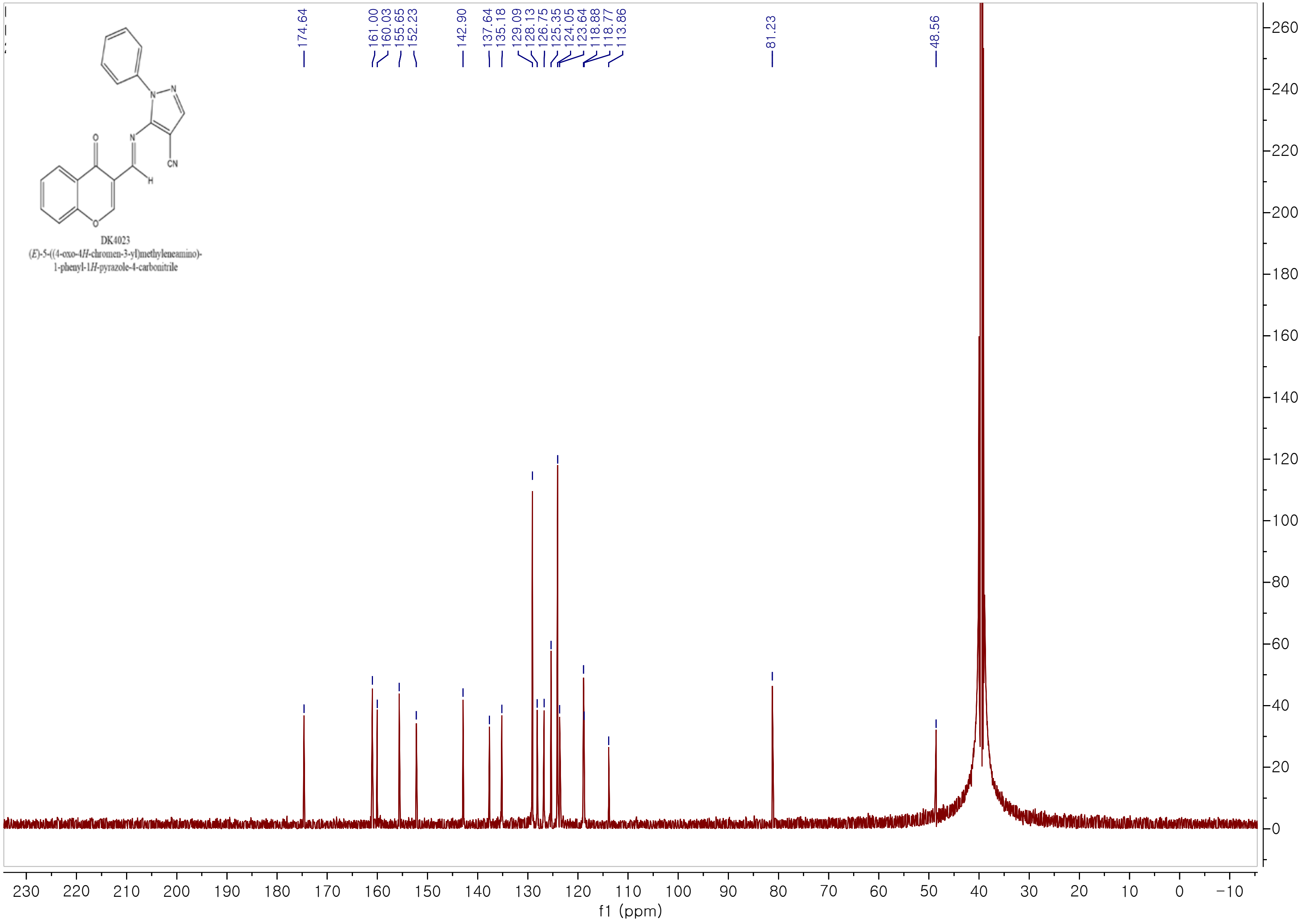

DK4023 HR-ESI-Mass spectrum

**Data Filename**	126-DK4023-2-n.d	**Sample Name**	126-DK4023-2-n
**Sample Type**	Sample	**Position**	P2-A3
**Instrument Name**	Instrument 1	**User Name**	
**Acq Method**	DirectMS-2min-n.m	**Acquired Time**	5/11/2020 11:25:13 PM
**IRM Calibration Status**	Success	**DA Method**	0829-re reaction.m
**Comment**			

**Sample Group**		**Info.**	
**Stream Name**	LC 1	**Acquisition SW Version**	6200 series TOF/6500 series Q-TOF B.06.01 (B6172 SP1)

**Data Filename**	60-DK4023-2-p.d	**Sample Name**	60-DK4023-2-p
**Sample Type**	Sample	**Position**	P2-A3
**Instrument Name**	Instrument 1	**User Name**	
**Acq Method**	DirectMS-2min-p.m	**Acquired Time**	5/11/2020 8:19:07 PM
**IRM Calibration Status**	Success	**DA Method**	0829-re reaction.m
**Comment**			

**Sample Group**		**Info.**	
**Stream Name**	LC 1	**Acquisition SW Version**	6200 series TOF/6500 series Q-TOF B.06.01 (B6172 SP1)

User Spectra

Peak List

m/z	z	Abund
187.08785	1	726727.69
217.09762	1	210406.41
230.09329	1	254178.34
341.1036	1	275154.25
363.08676	1	3702572.25
364.08935	1	699664.31
395.11268	1	3075060.75
396.11593	1	647629.63
633.18296	1	166180.47
922.0098	1	124591.43

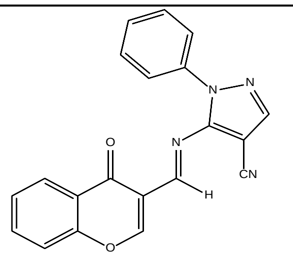

DK4023
(*E*)-5-((4-oxo-4*H*-chromen-3-yl)methyleneamino)-1-phenyl-1*H*-pyrazole-4-carbonitrile
Chemical Formula: $C_{20}H_{12}N_4O_2$
Exact Mass: 340.0960
Molecular Weight: 340.3420

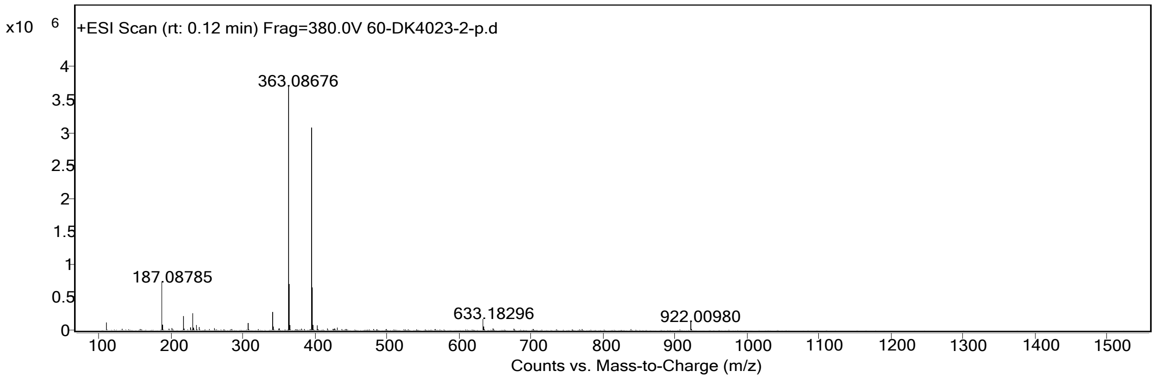

Supplement: Supplementary file 1 [file ijms-21-05080-s001.pdf]
